# Exploring the performance of implicit neural representations for brain image registration

**DOI:** 10.1038/s41598-023-44517-5

**Published:** 2023-10-13

**Authors:** Michal Byra, Charissa Poon, Muhammad Febrian Rachmadi, Matthias Schlachter, Henrik Skibbe

**Affiliations:** 1 RIKEN Center for Brain Science, Wako, Japan; 2 Institute of Fundamental Technological Research, Polish Academy of Sciences, Warsaw, Poland; 3 Faculty of Computer Science, University of Indonesia, Depok, Indonesia

**Keywords:** Neuroscience, Engineering, Biomedical engineering, Computational science, Image processing, Machine learning

## Abstract

Pairwise image registration is a necessary prerequisite for brain image comparison and data integration in neuroscience and radiology. In this work, we explore the efficacy of implicit neural representations (INRs) in improving the performance of brain image registration in magnetic resonance imaging. In this setting, INRs serve as a continuous and coordinate based approximation of the deformation field obtained through a multi-layer perceptron. Previous research has demonstrated that sinusoidal representation networks (SIRENs) surpass ReLU models in performance. In this study, we first broaden the range of activation functions to further investigate the registration performance of implicit networks equipped with activation functions that exhibit diverse oscillatory properties. Specifically, in addition to the SIRENs and ReLU, we evaluate activation functions based on snake, sine+, chirp and Morlet wavelet functions. Second, we conduct experiments to relate the hyper-parameters of the models to registration performance. Third, we propose and assess various techniques, including cycle consistency loss, ensembles and cascades of implicit networks, as well as a combined image fusion and registration objective, to enhance the performance of implicit registration networks beyond the standard approach. The investigated implicit methods are compared to the VoxelMorph convolutional neural network and to the symmetric image normalization (SyN) registration algorithm from the Advanced Normalization Tools (ANTs). Our findings not only highlight the remarkable capabilities of implicit networks in addressing pairwise image registration challenges, but also showcase their potential as a powerful and versatile off-the-shelf tool in the fields of neuroscience and radiology.

## Introduction

Pairwise image registration techniques designed to spatially align images are essential for various tasks in biomedical imaging. The goal of the registration is to determine a spatial transformation that maximizes the similarity between the moving image and the target fixed image. Accurate image registration enables data comparisons across subjects, imaging modalities and time. For example, registration is necessary in neuroscience to map an individual brain image to a population based brain atlas space.

Image registration methods have been studied for many years. Classic pair-wise image registration techniques, available in the Advanced Normalization Tools (ANTs) or NiftyReg, utilize handcrafted optimization algorithms to determine the deformation field^1,2^. Traditional methods can be used to produce well-behaved deformation fields that follow imposed modelling assumptions, for example, that the transformation function is affine or rigid. Classic methods can also be applied for deformable image registration, where the aim is to calculate a dense and nonlinear mapping between the two images. Unfortunately, classic algorithms can be time consuming for large 3D images and may underperform for data that violate field modeling assumptions.

Deep learning methods based on convolutional neural networks are gaining momentum in image registration^3^. Convolutional networks, such as VoxelMorph, can be used to process a pair of input images and output the deformation field required for image alignment^4^. Various deep learning methods based on the VoxelMorph framework have been proposed to further improve image registration performance. For example, TransMorph replaces the standard convolutional layers with transformer blocks^5^. DiffuseMorph determines the deformation field based on a conditioned diffusion process^6^. CycleMorph utilizes the cycle consistency loss to regularize the deformation field^7^. However, deep learning methods based on the VoxelMorph framework exhibit a number of limitations that diminish their practical applicability. First, such neural networks require large training sets and commonly underperform on out-of-distribution data. In practice it is often difficult to collect and curate a large dataset of biomedical imaging data. Additionally, utilizing convolutional networks for training with large input images demands significant memory resources, necessitating the downsampling of images to a predetermined geometry. This constraint complicates the network’s application to images that deviate from the training geometry. Furthermore, compared to traditional methods, VoxelMorph-like networks cannot serve as off-the-shelf tools for image registration, especially in neuroscience where the experimental data are scarce and commonly obtained using different acquisition protocols. SynthMorph has been proposed recently to address several limitations of VoxelMorph^8^. SynthMorph is trained with synthetic pairs of images, where a random deformation transformation is applied to cause dis-similarity between the input images. While this approach mitigates the requirement for the collection and curation of biomedical data, it does neither address the problems related to the image geometry, nor the problem with out-of-distribution image data, especially for cases requiring deformation fields that are different from the ones used to generate the synthetic training data.

Implicit neural representations (INRs) have recently been used for image registration in magnetic resonance imaging (MRI) and computed tomography (CT)^9,10^. INRs serve as a continuous, coordinate-based approximation of the deformation field obtained through a multi-layer perceptron (MLP). In this setting, a single implicit network is trained to directly determine the deformation field required to register two images. INRs offer a hybrid approach to image registration that presents properties of both neural networks and traditional per-case optimization techniques. Unlike VoxelMorph-like networks, INR based registration methods do not require large training datasets, as the implicit network is trained from scratch for each image pair. Moreover, compared to convolutional networks that require dense inputs (e.g. entire images) defined on coordinate grids with a predetermined size, implicit networks are defined on continuous coordinate spaces. This makes them suitable for the registration of images that differ in geometry and spatial resolution. Compared to traditional registration methods, implicit networks can leverage complex objective loss functions during the optimization. Wolterink et al. presented that implicit sinusoidal representation networks (SIRENs) can be used for lung registration in CT images^10^. The authors demonstrated that SIRENs outperform networks utilizing the rectified linear unit (ReLU) activation function. To perform the registration, the authors trained the implicit network with the standard normalized cross-correlation loss and randomly sampled image pixel coordinates. Similarly, Sun et al. successfully applied standard SIREN models for brain image registration in MRI^9^. The authors trained the implicit network based on the local normalized cross-correlation loss using a sampling strategy that utilizes randomly extracted 3D coordinate vector patches. Both studies demonstrated that INRs can be successfully used to model deformation fields with deep learning libraries that support GPU computations and automatic differentiation techniques, highlighting the potential of implicit networks as versatile off-the-shelf tools for paired image registration.

In this work, we further explore the use of implicit networks for brain image registration. Our main contributions are as follows:

- We investigate the registration performance of implicit networks equipped with activation functions that have different oscillatory characteristics. We propose several novel activation functions, such as the chirp function.
- We examine the performance of the INR based registration methods in several ways. For example, we investigate the trade-off between the registration performance and deformation field folding. Moreover, we investigate the relationship between the registration performance and oscillatory characteristics of the activation functions. Our results indicate several practical challenges in the application of implicit networks, such as the requirement for the hyper-parameter tuning.
- We propose novel INR based registration approaches that outperform the standard technique based on the SIREN network. For example, we present that the registration performance of the implicit networks can be improved by the incorporation of the cycle consistency loss. We also consider ensembles and cascades of implicit networks. Furthermore, we show that image registration can be combined with image fusion in the INR framework. Implemented methods are compared with the ANTs registration algorithm and VoxelMorph convolutional network.

## Methods

### Preliminaries

**Figure 1:**
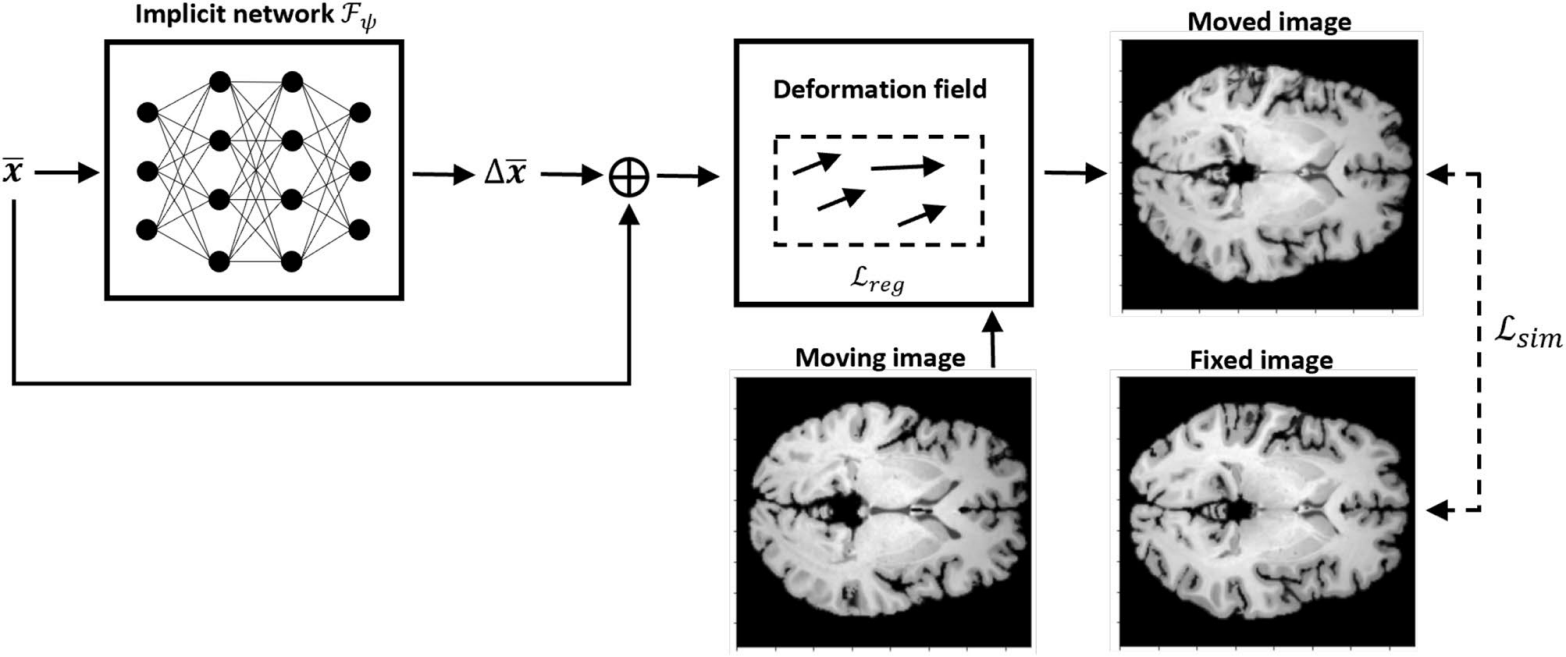
Scheme presenting pairwise brain image registration based on implicit neural representations. In this setting, a coordinate network is used to estimate the displacement vector $\Delta {\bar{x}}$ and determine the deformation field. Note changes in the shape of the brain, gyri, and ventricles in the moved image compared to the moving image.

The goal of pairwise 3D image registration is to determine a transformation $T_{\Phi }$ that spatially aligns a moving image $M:{\mathbb {R}}^3 \rightarrow {\mathbb {R}}$ to a fixed image $F:{\mathbb {R}}^3 \rightarrow {\mathbb {R}}$. Coordinate-based MLPs serve as networks that implicitly learn the transformation $T_{\Phi }$ by approximating the underlying deformation field, represented by $\Phi : {\mathbb {R}}^3 \rightarrow {\mathbb {R}}^3$. This field determines the displacement of the moving image *M* to spatially align it with the fixed image *F*^10^. We normalize coordinates with respect to the image dimensions so that all coordinates are within $[-1,1]^3$. In this setting, a single implicit network ${\mathscr {F}}_{\psi }$ with weights $\psi$ is used to process 3D spatial coordinates ${\bar{x}}\in [-1,1]^3$ of the moving image to output the displacement vector $\Delta {\bar{x}} \in {\mathbb {R}}^{3}$. Following the calculations of the displacement vectors, the deformation field is determined as $\Phi ({\bar{x}})= {\bar{x}} + \Delta {\bar{x}}$ and an interpolation algorithm is applied to obtain the corresponding moved image $T_{\Phi }(M)$. INR based registration is illustrated in Fig. 1.

In pairwise image registration, the following loss function is commonly used to determine the deformation field:

1 $$\begin{aligned} {\mathscr {L}}_{}(F, T_{\Phi }(M)) = {\mathscr {L}}_{sim}(F, T_{\Phi }(M)) + \alpha _{reg} {\mathscr {L}}_{reg}(\Phi ), \end{aligned}$$

where $\mathcal{L}_{sim}(F, T_{\Phi}(M))$ is the loss function designed to assess the similarity between the fixed image $F$ and the moved image $T_{\Phi}(M)$. $\mathcal{L}_{reg}(\Phi(\bar{x}))$ stands for the regularization loss function used to smooth the deformation field and to constrain the spatial transformation to be physically plausible. Moreover, $\alpha_{reg}$ indicates the weighting term used to balance the similarity and field regularization loss functions.

### Activation functions

Wolterink et al. demonstrated that SIRENs, MLPs equipped with the sine activation function, outperform ReLU networks in CT image registration^10^. In this work, we compare the performance of the sine and ReLU activation functions in more detail. In addition, we investigate the use of several other activation functions with frequency dependent components, listed in Table 1.

#### ReLU

ReLU became the default activation function for many deep neural networks because of its simplicity and satisfactory performance. It is defined by:

2 $$\begin{aligned} \sigma (x) = \text {max}(0, x). \end{aligned}$$

#### Sine

A common network architecture for modeling signals is the SIREN architecture^11^, which utilizes the sine activation function:

3 $$\begin{aligned} \sigma (x) = \text {sin}(\omega x), \end{aligned}$$

where *x* denotes the input, and $\omega$ represents the frequency-related hyper-parameter, typically set to 30 in applications^11^. The sine function addresses several problems associated with the ReLU function, such as the absence of higher-order derivatives and a bias towards low-frequency contents that can hinder the modeling of fine details in the underlying signals^11,12^. Additionally, networks with activation functions that possess periodic inductive biases are better suited to model functions with oscillating patterns^13^.

#### Snake

Ziyin et al. introduced the snake activation function as an alternative to the sine function, demonstrating that networks using the snake function are easier to optimize and can show superior performance in classification and sequence modeling tasks^13^. The snake function combines the identity mapping with a scaled cosine function:

4 $$\begin{aligned} \sigma (x) = x + \frac{1}{\omega } \text {cos}(\omega x) + \frac{1}{\omega }. \end{aligned}$$

#### Sine+

Sine+ is another function proposed by Ziyin et al. that shares similar advantages with the snake function when compared to the sine function^13^, defined as:

5 $$\begin{aligned} \sigma (x) = x + \text {sin}(\omega x). \end{aligned}$$

#### Chirp

We explore a chirp activation function as an alternative to the sine function. The sine function’s frequency is constant, controlled by the $\omega$ hyper-parameter, whereas the chirp function modulates with respect to frequency, and is therefore potentially better suited for oscillatory patterns of varying frequencies. We propose using the following chirp function as the activation function:

6 $$\begin{aligned} \sigma (x) = \text {sin}(\omega x + a_{\omega } x \text {tanh}(\omega x)), \end{aligned}$$

where $a_{\omega }$ is a hyper-parameter related to $\omega$, which constrains the rate of change in frequency with respect to the output of the hyperbolic tangent function. This way the frequency is variable for *x* values around 0 and approach constant values otherwise.

#### Morlet

We also explore the potential of the Morlet wavelet as an alternative to the sine function. The Morlet wavelet is a windowed modulated sine function, which, due to its locality in both spatial and frequency domains, may better adapt frequencies based on the location within the image. For simplicity, we use the imaginary part of the Morlet wavelet as the activation function^14^:

7 $$\begin{aligned} \sigma (x) = a_{\omega } e^{-\frac{1}{2}x^2}(sin(\omega x) - b_{\omega }), \end{aligned}$$

where $$a_{\omega } = \pi ^{-\frac{1}{4}} \left( 1+e^{-\omega ^{2}}-2e^{-\frac{3}{4}\omega ^{2}}\right) ^{-\frac{1}{2}}$$ and $$b_{\omega }=e^{-\frac{1}{2}\omega ^{2}}$$.

**Figure 2:**
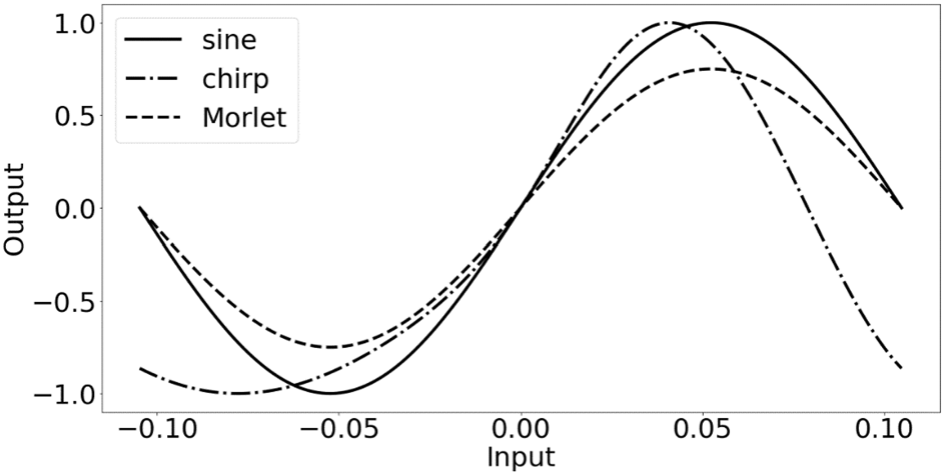
Sine, chirp and Morlet activation functions around 0 for $\omega$ set to 30. For the chirp function the hyper-parameter $a_{\omega }$ was set to 10.

Figure 2 shows the chirp and Morlet activation functions for inputs around 0.

**Table 1:** Investigated activation functions.

Name	$\sigma (x)$
ReLU	max(0, *x*)
sine	sin($\omega x$)
snake	$x + \frac{1}{\omega } \text {cos}(\omega x) + \frac{1}{\omega }$
sine+	$x+$sin($\omega x$)
chirp	$\text {sin}(\omega x + a_{\omega } x \text {tanh}(\omega x))$
Morlet	$a_{\omega } e^{-\frac{1}{2}x^2}(\text {sin}(\omega x) - b_{\omega })$

$a_{\omega }$ and $b_{\omega }$ indicate constants that depend on the frequency related hyper-parameter $\omega$.

### Investigated methods

In this section, we describe several potential extensions of the standard INR based approach to 3D image registration.

#### Attention mechanism

In the regular SIREN architecture, the $\omega$ hyper-parameter is constant for each linear layer. Since $\omega$ is related to the ability of the network to represent local signal variations, it may be beneficial to express this parameter as a function of the input coordinate ${\bar{x}}$, allowing the network to adjust the $\omega$ hyper-parameter according to local field characteristics. To achieve this, we propose a simple attention mechanism and modify the sine activation function in the following way:

8 $$\begin{aligned} \sigma (x) = \text {sin}(\omega x) = \text {sin} \big ( (\omega _0 + \Delta \omega f(x) ) x \big ), \end{aligned}$$

where $\omega _0$ and $\Delta \omega$ correspond to the center and modulation parameters, respectively. Function *f*(*x*) is used to relate the $\omega$ hyper-parameter to the output of the linear layer and has the following form:

9 $$\begin{aligned} f(x) = \text {tanh} \left( \frac{a}{n} \sum _{i=1}^n |x_i| + b \right) , \end{aligned}$$

where *a* and *b* are trainable parameters used for scaling, tanh is the hyperbolic tangent activation function and the summation goes over all units of the corresponding linear layer. We use the average absolute input values because we expect that coordinates associated with larger deformations produce higher outputs through the linear layers of the network. We set the $\omega _0$ and $\Delta \omega$ to 30 and 20, therefore the possible range for the $\omega$ hyper-parameter in Eq. (8) is equal to (10, 50).

#### Ensemble of implicit networks

Model ensembling is a popular strategy used to improve performance in machine learning^15^. We investigate whether an ensemble of three implicit networks can enhance the registration performance.

We create an ensemble based on three standard SIREN models with the $\omega$ hyper-parameters set to 10, 30, and 50, respectively, spanning a wide frequency range. Thus, the ensemble should model deformation fields with varying local variability. We generate the deformation field by averaging the output of all three networks, and train the networks jointly.

#### Affine transformation

Coordinate-based MLPs can be extended and jointly optimized with other image registration objectives. We present an INR that combines local deformable INR-based registration with a global image transformation by jointly training it with an affine transformation^16^. In this case, the deformation field can be expressed as follows:

10 $$\begin{aligned} \Phi _A({\bar{x}}) = A(M){\bar{x}} + t(M) + \Delta {\bar{x}}, \end{aligned}$$

where $\Delta {\bar{x}}$ is the displacement vector calculated with the implicit network, and the matrix $A(M) \in {\mathbb {R}}^{3\times 3}$ and the translation vector $t(M) \in {\mathbb {R}}^{3}$ represent the 12 trainable parameters for the global affine transformation. For training, *A*(*M*) and *t*(*M*) are initialized as the identity transformation.

#### Cascade of implicit networks

Cascading learning is another strategy used to enhance the performance of machine learning models. In a cascade, each model is trained from scratch, one after another, to correct and improve its predecessor’s outcome^17,18^. Due to its computational cost, this strategy is not often used with deep learning models. Instead, Vos et al. and Zhao et al. employed small multi-stage convolutional networks for image registration^19,20^, which were trained jointly in a single training episode. In this study, we investigate two-stage cascades of implicit networks for image registration. We develop a single implicit network for registration and train a second network to subsequently improve the deformation field. This approach can be expressed as follows:

11 $$\begin{aligned} \Phi _C({\bar{x}}) = \Phi ({\bar{x}}) + \Delta {\bar{x}}', \end{aligned}$$

where $\Phi ({\bar{x}})$ is the deformation field calculated by the first network and $\Delta {\bar{x}}'$ is the displacement vector estimated by the second network. For the first network we use a standard implicit model. For the second network, we examine two approaches. First, we train another regular model. Second, we train the second model with a higher regularization loss weight $\alpha$ to locally smooth the deformation field outputted by the first network.

#### Cycling implicit networks

The cycle consistency loss has found application in deep learning, particularly for tasks like image-to-image translation^21^. Kim et al. showcased that it can be used to enhance the performance of VoxelMorph through deformation field regularization^7^. In their framework, two convolutional networks are employed for image registration: the first network registers the moving image to the fixed image, and the second network does the reverse. After this initial registration, the roles of the networks are swapped, and the previously transformed images are registered once more. As a result, by the end of the two rounds of registration, the images should revert to their original spaces. The goal of introducing the cycle consistency loss is to enhance topological preservation. This idea of the symmetric normalization is not novel; it has been a crucial component of traditional image registration frameworks^22^. Drawing inspiration from this, our study introduces an analogous approach. Given that implicit networks operate on coordinates, we use coordinate specific displacement vectors to define the consistency loss. Let ${\mathscr {F}}_{M}$ and ${\mathscr {F}}_{F}$ stand for the implicit networks trained to transform the moving and the fixed image, respectively. The proposed cycle consistency loss is based on the following equations:

12 $$\begin{aligned} {\mathscr {L}}_{MF}({\bar{x}})&= \bigl ({\mathscr {F}}_{F}({\mathscr {F}}_{M}({\bar{x}})+{\bar{x}}\bigl ) + {\mathscr {F}}_{M}({\bar{x}}))^2, \end{aligned}$$

13 $$\begin{aligned} {\mathscr {L}}_{FM}({\bar{x}})&= \bigl ({\mathscr {F}}_{M}({\mathscr {F}}_{F}({\bar{x}})+{\bar{x}}) + {\mathscr {F}}_{F}({\bar{x}}) \bigl )^2,\end{aligned}$$

14 $$\begin{aligned} {\mathscr {L}}_{cyc}({\bar{x}})&= \frac{1}{2} \bigl ( {\mathscr {L}}_{MF}({\bar{x}}) + {\mathscr {L}}_{FM}({\bar{x}}) \bigl ), \end{aligned}$$

which promote correspondence between the displacement vectors. Next, we add the cycle consistency loss function ${\mathscr {L}}_{cycle}({\bar{x}})$ to the registration loss function in Eq. (1):

15 $$\begin{aligned} {\mathscr {L}}_{}(F, T_{\Phi }(M)) = {\mathscr {L}}_{sim}(F, T_{\Phi }(M)) + \alpha _{reg} {\mathscr {L}}_{reg}(\Phi ) + \alpha _{cyc} {\mathscr {L}}_{cyc}({\bar{x}}), \end{aligned}$$

where $\alpha _{cycle}$ indicates the loss weighting parameter.

#### Registration guided image fusion

**Figure 3:**
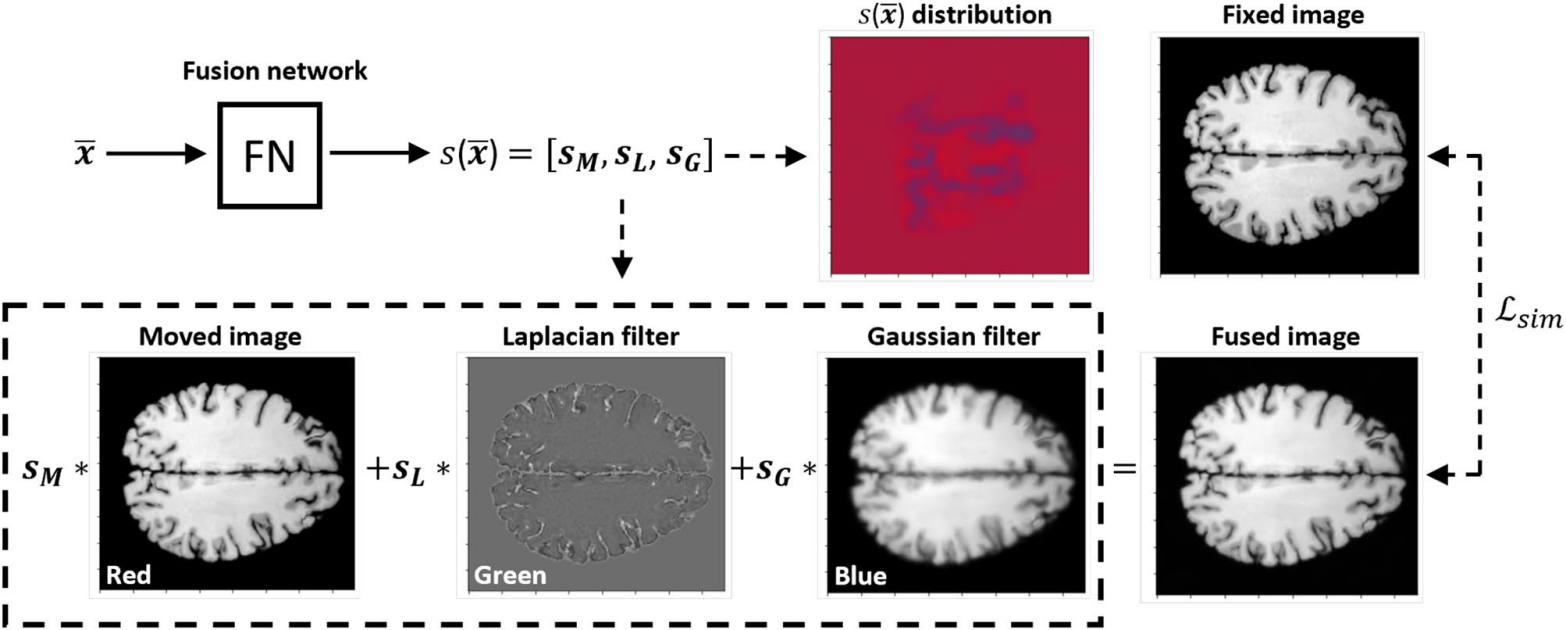
Scheme illustrating the proposed registration guided image fusion technique. The fused image is generated based on the weighted pixel-wise combination of the original image and images processed with the Laplacian and Gaussian filters. Weights used for image fusion are calculated using an implicit network trained to improve the registration. The weights of the distribution $s({\bar{x}})=[s_M({\bar{x}}),s_L({\bar{x}}),s_G({\bar{x}})]$ can be visualized with a parametric map, which pixel color intensities in the RGB space are proportional to each component of the distribution. For instance, the first weight, $s_M({\bar{x}}) \in [0,1]$, was mapped to [0, 255] in the red color channel. The parametric map indicates the filtering operations used to process particular image area.

In computer vision, implicit networks have been used to jointly perform several tasks, for example image reconstruction and style transfer^23,24^. In this study, we demonstrate that implicit networks can be used to connect brain image registration with the image fusion task. The proposed method is illustrated in Fig. 3. Various custom image processing algorithms have been designed to improve image registration. For example, for some applications it is common to utilize edge enhancing filters to highlight tissue contours, thereby guiding the registration algorithm to better match the edges between the moving and fixed images^25^. On the contrary, image smoothing filters have been also applied to improve the registration^26^. For example, the Gaussian filter can be used to process the moving image to remove local noise patterns that confound the similarity loss function designed to assess the alignment between the moving and fixed images. We develop an implicit network to combine these two image pre-processing approaches, namely edge enhancing filters and image smoothing filters, in an automatic and registration guided way. The proposed method can be used to fuse the edge enhanced and smoothed images in a coordinate-wise manner, enhancing the edge information in particular regions while smoothing the other areas. We formulate the image fusion problem in the following way:

16 $$\begin{aligned} M'({\bar{x}}) = s_M({\bar{x}}) M({\bar{x}}) + s_L({\bar{x}}) M_L({\bar{x}}) + s_G({\bar{x}}) M_G({\bar{x}}), \end{aligned}$$

where $M_L$ and $M_G$ stand for the moving images filtered with the Laplacian and Gaussian filters, respectively. Weights $s({\bar{x}})=[s_M({\bar{x}}),s_L({\bar{x}}),s_G({\bar{x}})]$ are used to fuse the corresponding images in a pixel-wise manner. In addition, we impose the following condition on the weights to constrain the fusion:

17 $$\begin{aligned} s_M({\bar{x}}) + s_L({\bar{x}}) + s_G({\bar{x}}) = 1. \end{aligned}$$

During the training of the deformation network, we jointly train a separate fusion network ${\mathscr {F}}_{\psi }^{fus}$ to output the weight vector $s({\bar{x}})$. For the fusion network, we use the standard SIREN model with the default $\omega$ parameter of 30. To train the network, we use the ${\mathscr {L}}_{fus}$ loss function based on the standard registration similarity loss, Eq. (1), which can be expressed in the following way:

18 $$\begin{aligned} {\mathscr {L}}_{fus}(F, T_{\Phi }(M), T_{\Phi }(M')) = \frac{1}{2} \left( {\mathscr {L}}_{sim}(F, T_{\Phi }(M)) + {\mathscr {L}}_{sim}(F, T_{\Phi }(M')) \right) , \end{aligned}$$

where ${\mathscr {L}}_{sim}$ stands for the standard image similarity loss function and $T_{\Phi }(M')$ is the transformed moved fused image. The aim of the above loss function is to jointly estimate the deformation field and provide means for the image fusion mechanism. Some regions for which the similarity score function underperform might be uplifted with the second component of the ${\mathscr {L}}_{fus}$ loss function. To take into account the fused image, the general registration loss function in Eq. (1) is modified in the following way:

19 $$\begin{aligned} {\mathscr {L}}_{}(F, T_{\Phi }(M)) ={\mathscr {L}}_{fus}(F, T_{\Phi }(M), T_{\Phi }(M')) + \alpha _{reg} {\mathscr {L}}_{reg}(\Phi ). \end{aligned}$$

### Evaluations

#### Mindboggle dataset

To evaluate the registration performance, we used the MindBoggle dataset, which consists of 101 labeled T1-weighted 3D brain MR images from five sources^27^. The dataset includes detailed manual segmentations for 62 cortical brain regions, with 31 regions in each of the the right and left hemispheres.

We used MR images pre-processed by the authors of the dataset. Pre-processing included affine alignment to a standard MNI template, skull stripping and re-sampling to volume spacing of 1 mm × 1 mm × 1 mm^27^. In addition, we cropped the 3D brain images to dimensions of 176 × 192 × 176 voxels. We excluded one brain scan, because a skull-stripped version was not available. Next, we manually screened all data and found 14 incorrectly aligned images, for which we re-run an initial affine registration. The participants, consisting of 53 males and 47 females, ranged in age from 19 years to 61 years, with a mean age of 28 years. Two brain scans served as fixed images, the remaining 98 cases were divided into training, validation and test sets with a split of 73, 5, 20, respectively. For the validation and test set, images were sampled uniformly from each of the five MindBoggle dataset subfolders, such that each Mindboggle dataset subfolder was equally represented in the validation and test sets. The remaining 3D images were included in the training set.

#### Implementation

Except for the activation functions, we used the same MLP architecture for all experiments. Each network included five fully connected hidden layers, each with 256 neurons. In addition, we used the Fourier mapping with six frequencies to encode the input coordinates^12^. We also concatenated the encoded coordinates with the middle layer of the network to form a residual connection. Weights of the networks were initialized depending on the utilized activation function following the original papers. For the chirp and Morlet activation functions, we initialized the weights in a similar way as for the SIREN model. Moreover, initial weights of the last linear layer of the implicit networks were sampled uniformly from [-0.0001, 0.0001] interval to ensure that only small displacement vectors are outputted by the networks during the first training epochs. AdamW optimizer with a learning rate of 0.0001 was used to train the networks^28^. Each model was trained on a single NVIDIA A100 GPU.

Due to the large dimensions of 3D images, implicit networks are commonly trained using 3D patches. In this work, we trained the networks with 3D patches of size 32 × 32 × 32^9^. For each epoch, we sampled 500 patches at random spatial locations. To assess the similarity between the moved and fixed images, we used the following loss function^9,10^:

20 $$\begin{aligned} {\mathscr {L}}_{sim}(F, T_{\Phi }(M)) = {\mathscr {L}}_{ncc}(F, T_{\Phi }(M)) + {\mathscr {L}}_{lncc}(F, T_{\Phi }(M)), \end{aligned}$$

where ${\mathscr {L}}_{ncc}$ indicates the normalized cross-correlation loss ($|1-\text {NCC}(F, T_{\Phi }(M))|$), calculated based on the entire 32 × 32 × 32 patch. ${\mathscr {L}}_{lncc}$ is the averaged local normalized cross-correlation loss computed using 9 × 9 × 9 windows over the 32 × 32 × 32 patch, determined to combine local and global alignment information. Following the previous study on the INR based registration, we used the following loss to condition the deformation field^10^:

21 $$\begin{aligned} {\mathscr {L}}_{reg}(\Phi ({\bar{x}})) = |1 - |J_{\Phi ({\bar{x}})}| |, \end{aligned}$$

where $|J_{\Phi ({\bar{x}})}|$ is the Jacobian determinant of the deformation field.

Calculations were done in Python using the PyTorch library^29^. Our implementations of the described methods are available at http://www.github.com/BrainImageAnalysis/INRsRegExp.

#### Performance assessment

The validation set was utilized to determine the better performing hyper-parameters. For the SIREN model, we examined five different $\omega$ values (10, 20, 30, 40, 50) and three $\alpha _{reg}$ values (1, 0.1, 0.01). Based on the validation set results we also determined the optimal number of training epochs. The SIREN models were compared with MLPs utilizing implemented activation functions, see Table 1. The $\alpha _{cyc}$ weight related to the cycle consistency loss, Eq. (15), was set to 100. Chirp rate $a_{\omega }$ was set to 10.

To assess the registration performance, we calculated the Dice scores between the manual segmentations of brain regions in the moved image and the fixed image^30^. The MindBoggle dataset consists of 62 annotated brain regions. First, we averaged the Dice scores calculated for each region. Second, to evaluate the performance for the most poorly aligned regions, we determined the lowest Dice score out of all 62 brain regions. Next, these two Dice scores obtained for each brain were averaged over all moving/fixed image pairs. To assess the deformation field folding, we calculated the percentage of non-positive Jacobian determinant values. We benchmarked the performance of our proposed methods against two established techniques: the ANTs symmetric image normalization (SyN) registration algorithm and the diffeomorphic VoxelMorph convolutional network^1,4,22,31^. For VoxelMorph, we constructed two networks using the training dataset, dedicating one for each fixed image. The networks were trained for 100 epochs using the same loss function as our implicit networks, Eq. (1). The regularization weights, denoted by $\alpha _{reg}$, were set to 0.1. For the SyN method, we optimized its parameters on the validation set with the objective to maximize the Dice score. The most optimal results were achieved using the cross-correlation loss function coupled with a three-level multi-resolution scheme. We allowed up to 500 iterations at the first level, 500 iterations at the second level and 500 iterations at the full resolution.

## Results

### Validation set experiments

**Figure 4:**
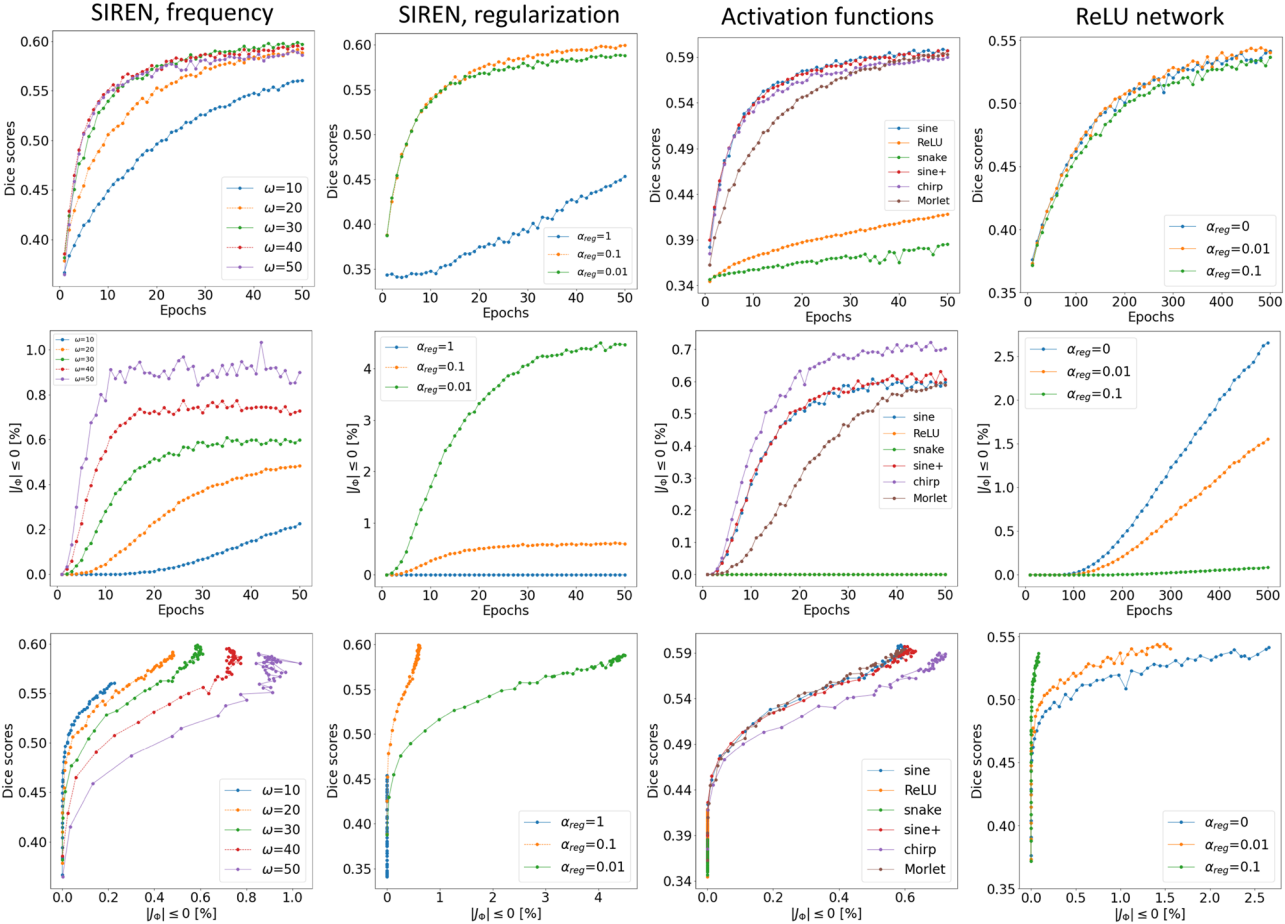
Registration performance scores obtained for the investigated implicit networks on the validation set. We found that the SIREN model trained with the $\omega$ hyper-parameter of 30 and $\alpha _{reg}$ of 0.1 performed best. Compared to the SIREN, network with the ReLU activation function required around ten more training epochs to reach performance plateu.

Figure 4 presents the validation set results obtained for the investigated methods. For the SIREN models trained with the regularization weight $\alpha _{reg}$ set to 0.1, we found that the value of the $\omega$ hyper-parameter affected the convergence characteristics and the deformation field properties. Similar Dice scores of around 0.57 were obtained for all investigated values of $\omega$ except for 10, for which the SIREN did not reach plateau for over 50 epochs. Deformation field folding was positively correlated with the value of the $\omega$ hyper-parameter, with the largest folding obtained for $\omega$ equal to 50. Moreover, the Spearman’s rank correlation coefficient between the Dice score and the percentage of the non-positive determinant values was equal to 0.92 for the SIREN network with $\omega$ set to 30. Overall, Fig. 4 suggests that $\omega$ equal to 30 provides well-balanced results with respect to convergence speed and deformation field folding. Setting $\omega$ equal to 30 also provided slightly better Dice scores on the validation set. For this case, the Dice score of the network plateaued after approximately 40 training epochs. Moreover, we also investigated the relationship between the registration metrics and the regularization weight $\alpha _{reg}$ obtained for the SIREN with $\omega$ set 30. We found that the regularization weight was important for image registration performance and deformation field characteristics, with $\alpha _{reg}$ equal to 0.1 providing good performance. For $\alpha _{reg}$ equal to 1, field regularization dominated the training and resulted in low Dice scores. On the contrary, $\alpha _{reg}$ of 0.01 was too small to address the problem of the deformation field folding.

Validation performance achieved by implicit networks equipped with different activation functions is illustrated in Fig. 4. In this case, the hyper-parameters $\omega$ and $\alpha _{reg}$ were set to 30 and 0.1 following the results from the previous paragraph. We found that networks with activation functions that have an oscillatory pattern, such as sine, sine+, chirp and Morlet, yielded comparable results. However, network with the snake activation function resulted in lower Dice scores. Due to the division of the cosine function by the $\omega$ parameter in the snake activation function (Eq. 4), the identity mapping probably dominated the activation function. The ReLU network did not reach plateau for over 50 epochs, therefore we trained it for up to 500 epochs, additionally examining three regularization weights $\alpha _{reg}$ of 0, 0.1 and 0.01, see Fig. 4. We found that network with the ReLU function required around 400 training epochs to reach plateau corresponding to the Dice score of around 0.53.

### Activation functions

Following the validation set experiments, we compared the test set performance of the implicit networks trained for 40 epochs with the $\omega$ hyper-parameter set to 30. The regularization weight $\alpha _{reg}$ was set to 0.1. Results are presented in Table 2. In addition, we also included the results for the network with the ReLU function trained for 400 epochs. We found that the networks utilizing ReLU and snake activation functions achieved worse performance scores compared to functions presenting oscillatory patterns. We obtained similar results for the sine, sine+, chirp and Morlet activation functions, confirming that the presence of the frequency component is important for the performance. Table 2 shows that the regular SIREN model achieved the highest Dice score of 0.576.

**Table 2:** Test set results (mean±std) on the MindBoggle dataset obtained for implicit networks equipped with different activation functions.

Method, epochs	Dice$_{\text {avg}}$ $\uparrow$	Dice$_{\text {min}}$ $\uparrow$	$|J_{\Phi }|\le$0 [%] $\downarrow$
sine, 40	0.576 ± 0.113	0.258 ± 0.097	0.673 ± 0.116
ReLU, 40	0.401 ± 0.098	0.169 ± 0.058	< 0.01
ReLU, 400	0.521 ± 0.103	0.225 ± 0.097	0.065 ± 0.035
snake, 40	0.360 ± 0.102	0.121 ± 0.050	< 0.01
sine+, 40	0.574 ± 0.112	0.253 ± 0.109	0.668 ± 0.115
chirp, 40	0.569 ± 0.112	0.258 ± 0.098	0.786 ± 0.131
Morlet, 40	0.570 ± 0.107	0.251 ± 0.097	0.620 ± 0.090

### Proposed methods

**Table 3:** Test results (mean±std) on the MindBoggle dataset obtained for the proposed INR based registration methods.

Method	Dice$_{\text {avg}}$ $\uparrow$	Dice$_{\text {min}}$ $\uparrow$	$|J_{\Phi }|\le$0 [%] $\downarrow$
None	0.325 ± 0.041	0.116 ± 0.047	–
ANTs SyN	0.544 ± 0.019	0.260 ± 0.103	< 0.01
Diffeomorphic VoxelMorph	0.539 ± 0.131	0.209 ± 0.077	0.202 ± 0.015
SIREN	0.576 ± 0.113	0.258 ± 0.097	0.673 ± 0.116
SIREN, trainable $\omega$	0.576 ± 0.111	0.254 ± 0.098	0.674 ± 0.113
SIREN, $\omega$ width modulation	0.564 ± 0.112	0.247 ± 0.099	0.787 ± 0.132
SIREN, attention	0.567 ± 0.110	0.256 ± 0.101	0.686 ± 0.187
SIREN and affine	0.576 ± 0.112	0.250 ± 0.102	0.682 ± 0.120
SIREN, ensemble	0.585 ± 0.113	0.255 ± 0.104	0.711 ± 0.117
SIREN, cycle consistency loss	0.578 ± 0.112	0.263 ± 0.105	0.372 ± 0.051
SIREN, cascade, $\alpha _{reg}=0.1$	0.594 ± 0.115	0.272 ± 0.101	0.666 ± 0.104
SIREN, cascade, $\alpha _{reg}=1$	0.579 ± 0.112	0.260 ± 0.099	0.123 ± 0.027
SIREN, fusion	0.579 ± 0.113	0.260 ± 0.106	0.648 ± 0.112

**Figure 5:**
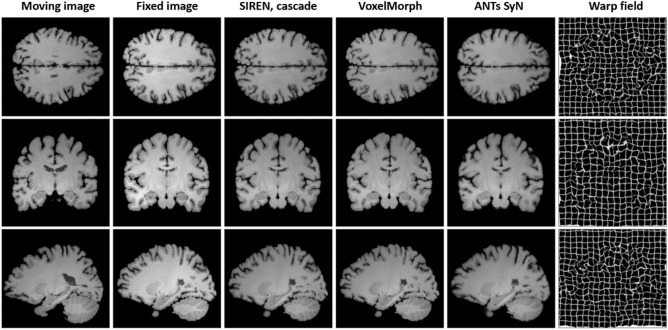
Qualitative comparison of the registration performance between the implicit networks, diffeomorphic VoxelMorph and ANTs SyN algorithm. The presented warp fields correspond to the implicit network.

**Figure 6:**
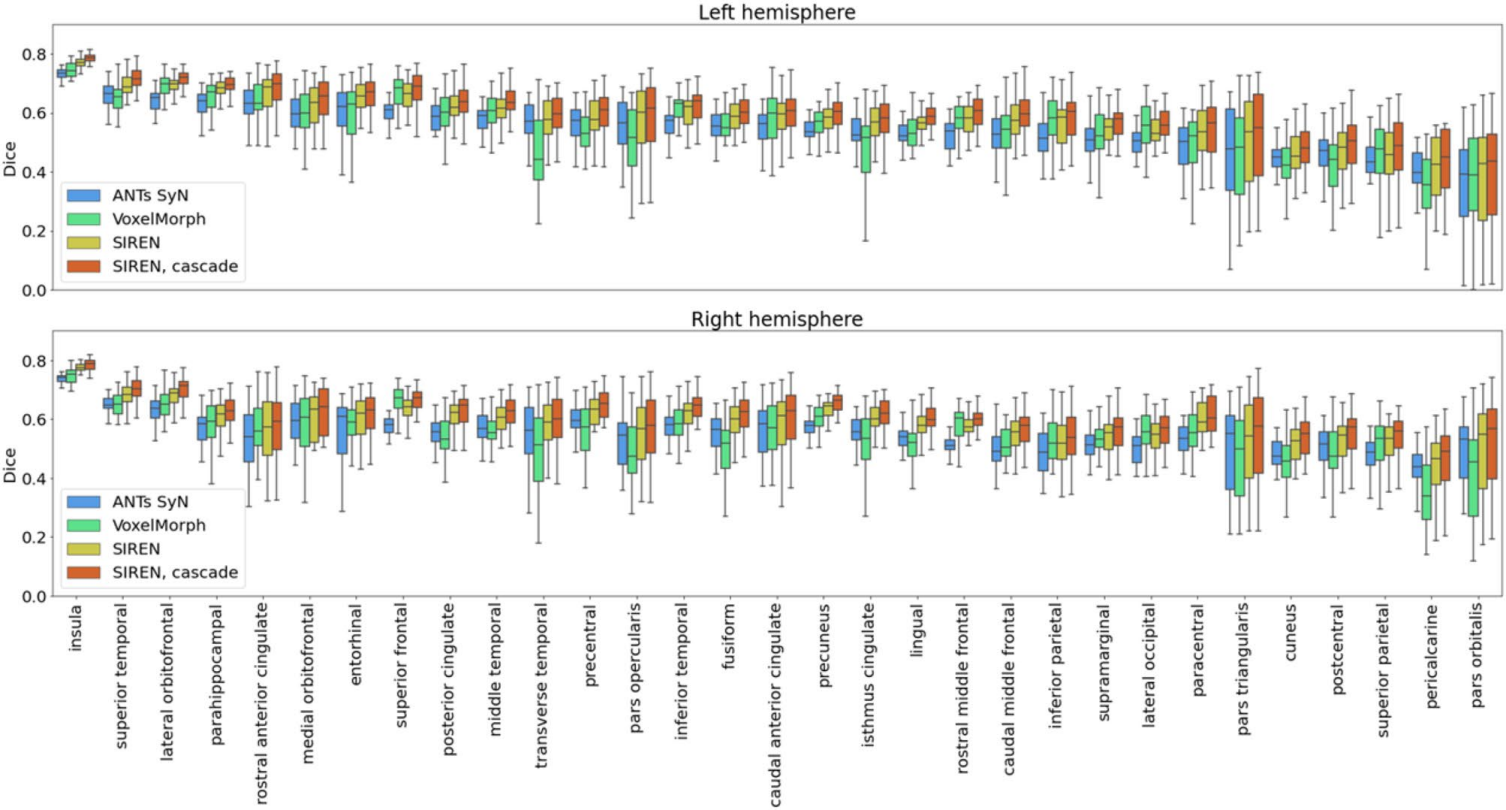
Boxplots illustrating Dice scores for various anatomical structures for image registration using ANTs SyN algorithm, diffeomorphic VoxelMorph, SIREN and cascade of SIRENs. Structures are ordered based on average ANTs Dice scores obtained for each structure in the left hemisphere. The approach based on the cascade of SIRENs achieved better performance than the other investigated methods.

Table 3 compares the performance of the standard methods and the techniques proposed to improve the INR based registration. A SIREN model with $\omega$ parameter set to 30 was used as the backbone for the proposed techniques. Following the validation set results, regularization weight $\alpha _{reg}$ was set to 0.1 and networks were trained for 40 epochs. For comparison, we also developed a SIREN model with a trainable $\omega$ hyper-parameter (initially set to 30) and a SIREN with the $\omega$ hyper-parameter modulated with respect to the layer width. In the latter case, for each layer unit of the network we linearly modified the $\omega$ values from 10 to 50. All methods based on implicit networks achieved higher Dice scores than the ANTs SyN optimization technique and the VoxelMorph. Figure 5 quantitatively compare registration performance of the selected techniques. In addition, Fig. 6 illustrates Dice scores for anatomical structures annotated in the MindBoggle dataset.

SIREN models with a trainable $\omega$ parameter achieved comparable scores to SIREN models with a fixed frequency parameter, suggesting that the $\omega$ value of 30 was optimally selected for the investigated registration task. Similarly, the utilization of the proposed attention mechanism or the modulation mechanism did not improve the performance compared to the SIREN with optimized $\omega$ hyper-parameter. This suggests that SIREN models with trainable $\omega$ can maintain the performance of the best networks with fixed parameters without the need for extensive hyper-parameter tuning.

**Figure 7:**
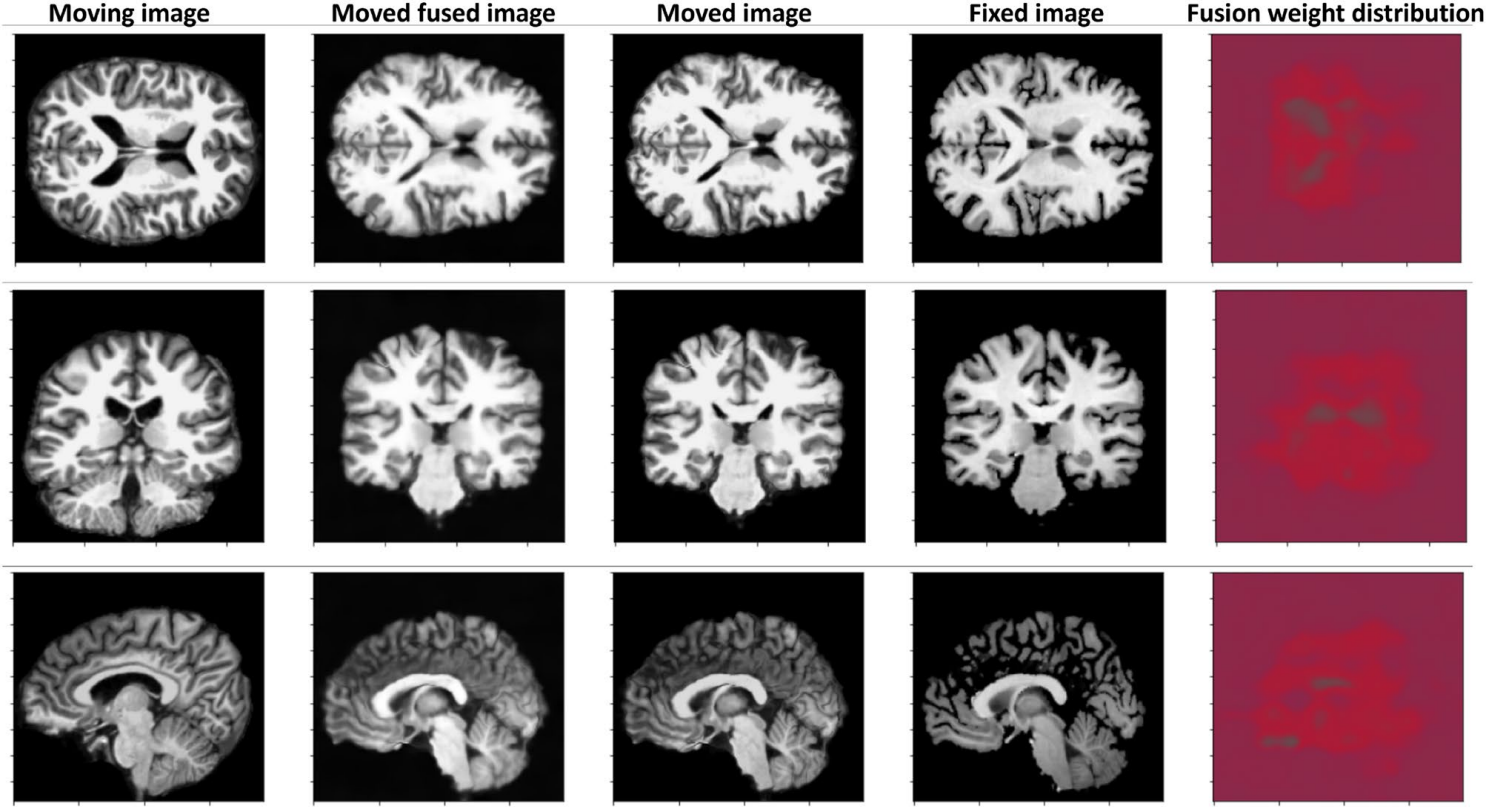
Sample results obtained for the implicit networks based on the proposed registration guided image fusion. Weight distribution indicates the utilization of the original image (red) and the Laplacian (green) and Gaussian (blue) filtered images. For this particular case, the average weights, Eq. (16), were equal to 0.57 (original), 0.15 (Laplacian) and 0.28 (Gaussian), indicating that all operations were utilized to process the brain image.

Method combining the trainable affine layer and the SIREN model achieved similar scores to the standalone SIREN model, presumably due to the accurate initial pre-alignment of the brain images to the standard MNI template space. Ensembling implicit networks achieved higher Dice score compared to the standard SIREN model. We found that utilization of the cycle consistency loss improved deformation field characteristics, decreasing the number of the folding pixels. Cascade of two identical SIREN models achieved the highest Dice score of 0.594. Cascade with the second network trained using $\alpha _{reg}$ equal to 1 resulted in the improvement of the deformation field. The method based on image fusion achieved slightly better performance than the standard SIREN model. Sample results for this approach are presented in Fig. 7. The weight distribution maps shows the automatic utilization of the image filtering operations. We found that the method utilized mainly the original image pixels with approximately 10% and 25% pixels extracted from the images processed by the Laplacian and Gaussian filters, respectively.

## Discussion

We presented several novel approaches to INR-based image registration. We conducted extensive experiments to show the impact of hyper-parameter selection and design choices on the performance of implicit networks. Our results demonstrated that implicit networks can achieve superior performance to traditional image registration methods (ANTs SyN) and standard deep learning image registration frameworks (VoxelMorph). In biomedical image analysis, traditional registration algorithms remain the go-to tools due to the diversity of complexity of the registration tasks. However, implicit networks, which combine the characteristics of standard optimization techniques with modern deep learning methodologies, offer a promising alternative. Unlike convolutional networks, which often demand extensive training data defined on fixed spatial grids, INR-based methods can function efficiently without such extensive datasets. Furthermore, for specific applications, implicit networks can be optimized on a case-by-case basis, potentially leveraging complex objective loss functions during the optimization. This adaptability also provides a solution to the out-of-distribution issues frequently encountered with large convolutional networks.

Presented validation set results illustrated that the choice of the hyper-parameters, such as the regularization weights, are important for the brain image registration performance. Optimization of model hyper-parameters based on the validation set is usually difficult to conduct with convolutional networks because the model must be retrained on the entire training dataset for each set of hyper-parameter values. Optimization of multiple hyper-parameters, each of which may affect another, may consume considerable time and computing resources^32^. In contrast, a small annotated validation set can be used to quickly explore the hyper-parameter space of implicit networks. Different combinations of hyper-parameters can also be utilized during the inference to generate a set of registered images for visual comparisons. Therefore, our results suggest that implicit networks are easier to optimize than methods based on convolutional networks, which should result in better registration capabilities.

We investigated image registration performance associated with six activation functions. To the best of our knowledge, our study demonstrated for the first time that the chirp function and the Morlet wavelet can be used as activation functions for implicit networks. However, we achieved similar registration scores for these two activation functions compared to the standard sine function. Our results indicated that the center frequency of the oscillations was the most important for the performance. Four activation functions, namely the sine, sine+, chirp and Morlet functions, achieved comparable registration performance. We presented that the frequency hyper-parameter $\omega$ is strictly related to the registration performance, with large values of $\omega$ correlated to the deformation field folding. Moreover, networks with activation functions presenting low-frequency contents, such as ReLU, were not suited to produce variable deformation fields. Network equipped with the ReLU function required many more training epochs to reach the performance score plateau compared to SIREN models. However, networks with ReLU require less hyper-parameter tuning, therefore might be attractive for some applications.

In this work, we examined several novel approaches to the INR based registration. Our findings demonstrated that implicit networks can serve as an efficient and flexible technique for pairwise brain image registration. First, it is possible to develop cascades of implicit networks to subsequently improve the deformation field and consequently the registration performance. While we only investigated cascades of implicit networks, the same approach can be applied to improve the deformation field determined by a convolutional network or a traditional optimization algorithm. Moreover, improvement of the deformation field can be restrained to a spatial region. Second, implicit networks can be easily used to form ensembles. For example, we trained the implicit network jointly with an affine transformation model. Third, since implicit networks can be trained with back-propagation, it is possible to associate the registration problem with other tasks. Here, we presented that INR based registration can be performed jointly with image fusion based on a second implicit network. The proposed INR based fusion method can provide some interpretability about the registration model, since it can be used to visualize image regions that require texture filtering for the registration. While we only utilized the Laplacian and Gaussian filters, any other operations can be easily considered within the proposed framework. The investigated INR based methods can also be combined. For example, it would be straightforward to combine the method based on image fusion with the technique utilizing the cycle consistency loss.

Our study presented several challenges associated with INR based registration methods. First, in our setting the implicit networks had to be trained from scratch for each image pair, slowing the inference time compared to standard convolutional networks. To address this problem, we would like to investigate methods that speed up computations. For example, it may be interesting to utilize hyper-networks to pre-initialize the weights of the implicit networks^33^. Second, while in this study we extensively explored the usefulness of the INRs for registration, our experiments were based on a single dataset. Although the MindBoggle dataset includes brain MR images from multiple sources and detailed annotations, it would also be interesting to utilize additional datasets for evaluation, e.g. computer tomography images or ultrasound data^34,35^. Third, we trained the networks with loss functions based on the cross-correlation function and the Jacobian determinant of the deformation field. To reduce potential biases in our experiments, we used the same network architecture and coordinate sampling procedure. Investigating other network architectures and training routines future studies would be interesting.

## Conclusion

In this work, we explored implicit neural representations for the registration of magnetic resonance brain images. We performed extensive experiments to compare different activation functions, including two novel functions proposed in this study: the chirp function and the Morlet wavelet. We also developed several novel implicit network based approaches to the registration, which outperformed the previously proposed method. Presented results indicate that implicit networks are well suited to address the problem of the pairwise image registration.

We believe that our work is an important pre-liminary step to a wider adoption of the implicit networks as a versatile off-the-shelf image registration tool. In the future, we plan to conduct additional experiments to further highlight the usefulness of the implicit networks for the image registration.

## Data Availability

All data used during this study are included in this published article and its supplementary information files: 10.3389/fnins.2012.00171, https://mindboggle.info/data^27^. Implementations of the investigated neural networks are available at http://www.github.com/BrainImageAnalysis/INRsRegExp.
